# Effects of Path-Finding Algorithms on the Labeling of the Centerlines of Circle of Willis Arteries

**DOI:** 10.3390/tomography9040113

**Published:** 2023-07-24

**Authors:** Se-On Kim, Yoon-Chul Kim

**Affiliations:** Division of Digital Healthcare, College of Software and Digital Healthcare Convergence, Yonsei University, Wonju 26493, Republic of Korea

**Keywords:** magnetic resonance angiography, cerebral arteries, vessel segmentation, graph structure, Dijkstra algorithm, A* algorithm, depth first search

## Abstract

Quantitative analysis of intracranial vessel segments typically requires the identification of the vessels’ centerlines, and a path-finding algorithm can be used to automatically detect vessel segments’ centerlines. This study compared the performance of path-finding algorithms for vessel labeling. Three-dimensional (3D) time-of-flight magnetic resonance angiography (MRA) images from the publicly available dataset were considered for this study. After manual annotations of the endpoints of each vessel segment, three path-finding methods were compared: (Method 1) depth-first search algorithm, (Method 2) Dijkstra’s algorithm, and (Method 3) A* algorithm. The rate of correctly found paths was quantified and compared among the three methods in each segment of the circle of Willis arteries. In the analysis of 840 vessel segments, Method 2 showed the highest accuracy (97.1%) of correctly found paths, while Method 1 and 3 showed an accuracy of 83.5% and 96.1%, respectively. The AComm artery was highly inaccurately identified in Method 1, with an accuracy of 43.2%. Incorrect paths by Method 2 were noted in the R-ICA, L-ICA, and R-PCA-P1 segments. The Dijkstra and A* algorithms showed similar accuracy in path-finding, and they were comparable in the speed of path-finding in the circle of Willis arterial segments.

## 1. Introduction

Abnormal morphological characteristics of the cerebral blood vessels are associated with vascular diseases. For example, stenosis of any artery due to intracranial atherosclerosis can cause ischemia in the brain tissue, resulting in stroke and other cognitive brain disorders [1,2]. Development of intracranial aneurysms [3,4] may be associated with high blood pressure on the weakened vessel wall and potentially could result in ruptures and cerebral hemorrhages if left untreated [5]. Three-dimensional (3D) computed tomography angiography (CTA) and 3D time-of-flight MR angiography (TOF-MRA) are commonly used to noninvasively obtain data on the cerebral vessels [6,7]. The geometric information such as vessel tortuosity [8] and distributions of the diameters of the vessels [9] is extracted and evaluated to study correlations between vessel abnormalities and diseases. Quantitative evaluation based on the geometry of the cerebral arteries has been reported in the literature [9,10,11]. For example, a cumulative distribution function of the cross-sectional diameters in a vessel segment was evaluated [9]. Tortuosity descriptors such as the sum of angle metrics, inflection count metric, triangular index, relative length, and product of angle distance were used to find the relationship between diseases and morphological features [12,13]. These quantities are typically calculated on a vessel segment after identifying the vessel’s centerline, and a path-finding algorithm can automate the identification of the centerline.

Labeling of the arteries in the circle of Willis (CoW) has been of interest to researchers since the CoW is essential in maintaining the circulation of blood flow even in the occlusion of an artery and has important bifurcations that anatomically separate the intracranial arteries of interest [14,15,16]. For example, the internal carotid artery (ICA) branches into the anterior cerebral artery (ACA) and middle cerebral artery (MCA). In the posterior circulation, the basilar artery (BA) is connected to the left and right posterior cerebral arteries (PCA). Tracking of the vessel’s centerline can be performed via a path-finding algorithm after manual annotations of two endpoints of a vessel segment. This procedure is relatively simple and straightforward when compared with deep learning-based segmentation of the intracranial vessel segments [17]. The development of deep learning segmentation models requires manual segmentations of individual arterial segments for the generation of training data. The manual segmentation process involves slice-wise manual tracing in a 3D volume consisting of hundreds of slices, and it is thus time-consuming and laborious [18]. Hence, the centerline tracking approaches can improve the efficiency of vessel labeling and its subsequent quantification without the need for deep learning model training data generation. Previous studies have demonstrated methods adopting path-finding algorithms such as Dijkstra’s algorithm in analyzing 3D angiography data [19,20,21]. However, they did not demonstrate detailed comparisons of accuracy among available path-finding algorithms in vessel image analysis.

In this study, we evaluated the performance of three path-finding algorithms in robustly identifying the cerebral arterial segments in the CoW. In terms of path-finding accuracy and computational time, we compared a depth-first search (DFS) based algorithm that does not involve a graph structure to a Dijkstra algorithm and an A* (A star) algorithm, both of which require a graph representation.

## 2. Materials and Methods

**Figure 1:**
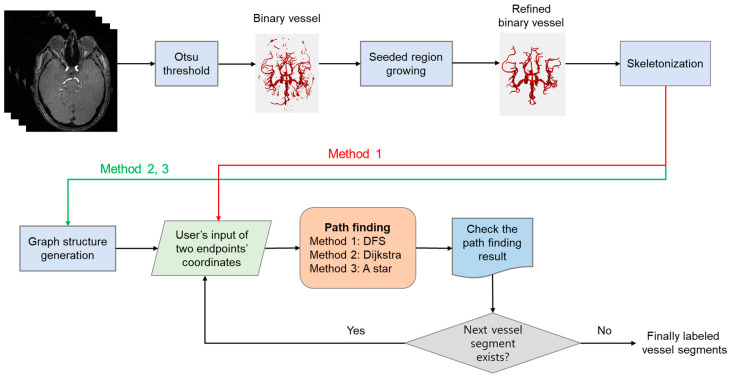
A flowchart of the segmental vessel-labeling algorithm. Three path-finding methods were used to label the segments in the circle of Willis. Notably, Method 1 does not require a graph structure generation process, while Methods 2 and 3 require the graph structure generation process.

Figure 1 illustrates the flowchart of the presented vessel labeling methods. The preprocessing steps involving Otsu thresholding and 3D seeded region growing have been implemented in Matlab version 9.13 (The Mathworks Inc., Natick, MA, USA). Skeletonization, graph structure generation, path-finding methods, and visualization of the centerlines in 3D were implemented in Python version 3.10 (Python Software Foundation).

### 2.1. Data

We used publicly available magnetic resonance angiography (MRA) data from the IXI Dataset (https://brain-development.org/ixi-dataset) (accessed on 21 July 2023). Sixty Neuroimaging Informatics Technology Initiative (NIfTI) files were considered to evaluate the performance of path-finding algorithms. The imaging parameters are as follows: Philips Medical Systems Intera 3T, repetition time = 16.7 ms, echo time = 5.8 ms, number of phase-encoding steps = 286, reconstruction diameter = 240 × 240 mm^2^, acquisition matrix = 288 × 286, flip angle = 16-deg, in-plane pixel spacing = 0.4–0.6 mm, spacing between slices = 0.8 mm. We applied the bi-cubic interpolation along the slice dimension to generate iso-resolution image data with the same pixel spacing in all three directions. After the interpolation, the final voxel size was isotropic with the 0.6 mm voxel spacing.

### 2.2. Vessel Segmentation and Skeletonization

The user selected an axial slice that shows three cross-sections of the vessels, which are the right internal carotid artery (ICA), left ICA, and basilar artery (BA). Supplementary Figure S1 shows an example axial slice showing three vessels’ cross-sections. A seed point was manually annotated by the mouse click within each of the three cross-sections (Supplementary Figure S1). With each seed point, 3D seeded region growing was performed using the region-growing function [22] available in Matlab. The segmented arteries after the region growing included the region of the CoW. The three segmentation results were obtained in binary masks, and the union set operation was performed to combine the three segmented masks into one final binary mask of the vessels. Skeletonization was performed to find the centerlines of the vessels using the scikit-image Python library [23].

### 2.3. Annotation of Two Endpoints

**Figure 2:**
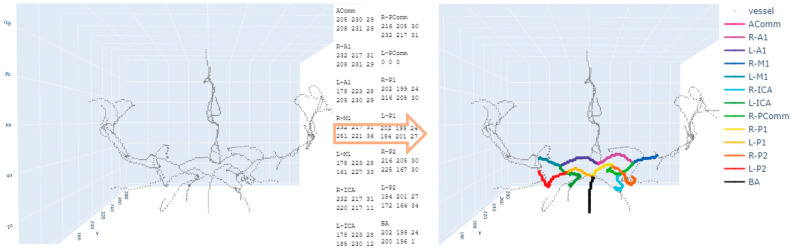
An example of colored labeling of the arteries in the circle of Willis. The **left** figure is the centerlines after skeletonization, and the **right** figure shows the labeled segments in colors after applying a path-finding algorithm. The numbers in the middle text show the 3D coordinates of the two endpoints in each vessel segment along with the vessel segment’s name.

Following the anatomical terminology provided by Dumais et al. [17], we identified 14 vessel segments by manually annotating two endpoints in each vessel segment. The vessel segments of interest were (1) AComm (anterior communicating artery), (2) R-A1 (right anterior cerebral artery A1), (3) L-A1 (left anterior cerebral artery A1), (4) R-M1 (right middle cerebral artery M1), (5) L-M1 (left middle cerebral artery M1), (6) R-ICA (right internal carotid artery), (7) L-ICA (left internal carotid artery), (8) R-PComm (right posterior communicating artery), (9) L-PComm (left posterior communicating artery), (10) R-P1 (right posterior cerebral artery P1), (11) L-P1 (left posterior cerebral artery P1), (12) R-P2 (right posterior cerebral artery P2), (13) L-P2 (left posterior cerebral artery P2), and (14) BA (basilar artery). Some endpoints were shared among the vessel segments. For example, the R-A1 and R-M1 segments share a common branch point (Figure 2). The same is true for the BA, R-P1, and L-P1 segments.

The Plotly (Plotly Technologies Inc., Montreal, QC, Canada) Python library (https://plotly.com/python) (accessed on 21 July 2023) was used to visually identify the vessel segments in the 3D space. The mouse hovering on the target endpoint shows its position information in 3D coordinates. For each vessel segment, the position information of the two endpoints was the input to our path-finding algorithms. During manual annotation, we recorded the position information of the two endpoints in vessel segments. If no centerline exists in a certain vessel segment, we recorded ‘0 0 0′ for the vessel segment. The position information was saved in a text file with .txt extension for each subject. The visualization result of the colored vessel segments was saved in a .html file, which was reserved for further investigation to check the accuracy of the vessel annotations.

### 2.4. Path-Finding Algorithms

In this study, we implemented three path-finding algorithms for comparisons: (Method 1) DFS-based path-finding algorithm, (Method 2) Dijkstra’s algorithm, and (Method 3) A* algorithm.

For Method 1, we implemented a maze-solving algorithm based on DFS (Supplementary Figure S2). Given two endpoints, from the start point, Method 1 attempted to find the next neighbor pixel and record the visited pixel locations using the push operation in a stack. When there is no neighboring pixel anymore, the visited locations were taken out of the stack until the algorithm found the neighboring pixel which was not visited. The algorithm was terminated when the endpoint was reached. The remaining data containing pixel locations in the stack indicate the coordinates along the path that connects the two endpoints. We implemented the method in C++ on the Microsoft Visual Studio environment. PyBind11 (https://github.com/pybind/pybind11) (accessed on 21 July 2023) was used to create Python bindings to the C++-compiled code [24]. Hence, the 3D coordinates along the path were able to be obtained and saved in a variable in Python.

Methods 2 and 3 require the generation of a graph structure from the skeleton vessel image as shown in Figure 1. To generate a graph from the skeleton image, we used the Skan version 0.10.0 Python library (https://skeleton-analysis.org/stable) (accessed on 21 July 2023) [25]. Dijkstra’s algorithm is well known as the shortest path-finding algorithm in a graph structure [26]. In our implementation, the pixels along the centerlines represented vertices (or nodes), and the connections with their neighboring pixels represented edges. The weights between the two connected nodes were calculated as the Euclidean distance of the two points. We used the Dijkstra() function provided by the SciPy Python library [27]. The Dijkstra() function is based on the Fibonacci heap implementation, which has the time complexity of O(E + V logV) (here, E is the number of edges, and V is the number of vertices) and is more time efficient than the list implementation whose time complexity is O($V^{2}$) [28]. A* algorithm is another method for the shortest path search in a graph structure. In contrast to Dijkstra’s algorithm, which finds the shortest path from a starting point to all goal points, the A* algorithm only finds the shortest path from a starting point to a destination point, and it introduces heuristic cost values as well as graph weights to find the next node [29]. In our study, we used and modified the code available from the python-astar open-source library (https://github.com/jrialland/python-astar) (accessed on 21 July 2023). The heuristic cost value was calculated by computing the Euclidean distance between the nodes of interest and the destination node.

### 2.5. Evaluation

We compared the three methods in terms of path-finding accuracy and computational time in Python. For each vessel segment, we provided the two endpoints and let the methods automatically find a path. We counted the number of incorrect paths and the number of correct paths for each vessel segment after building consensus on the path-finding correctness. An incorrect path was defined as a path that connects the two endpoints but is not the path we expected. To measure computational time, we used the time.perf_counter() function. Since we used the already annotated endpoints, during the time measurements there were no manual annotation procedures for recording two endpoints in all the vessel segments. For Method 1, we measured the time interval of the DFS path-finding algorithm taken to find paths in all segments in each subject. For Methods 2 and 3, we first measured the time interval of the graph structure generation from the skeleton image and then measured each time interval of the path-finding algorithm (i.e., SciPy’s Dijkstra() function for Method 2 and python-astar’s find_path() function for Method 3). For each method of Methods 2 and 3, we summed the time intervals including the graph structure generation and the path finding. These time intervals were compared to the time measured from Method 1 for each subject’s data. The evaluation was performed on a Windows PC (13th Generation Intel^®^ Core™ i7-13700K 16-Core Processor Central Processing Unit).

A Fisher’s exact test was used to analyze any differences between the path-finding methods in detecting the correct centerlines of the vessel segments. A two-sample unpaired Student’s *t*-test was performed to determine if the computational time between the two methods was significantly different. A *p*-value of <0.05 was considered statistically significant.

## 3. Results

Table 1 summarizes the accuracy of the three algorithms in finding the paths between two endpoints in each vessel segment. Method 1 produced 121 incorrect paths out of 735 paths, and Method 2 produced only 21 incorrect paths out of 735 paths. Method 3 produced 29 incorrect paths. The accuracy (97.1%) of Method 2 was significantly higher than that (83.5%) of Method 1. The accuracy of Method 3 was 96.1%. The AComm is highly inaccurate in Method 1 with an accuracy of 43.2%. Method 2 is superior to Method 1 in all vessel segments except for the R-ICA segment. Method 3 is comparable to Method 2 in every vessel segment, except for the AComm segment. Fisher’s exact test resulted in statistically significant associations in most vessel segments in Method 1 and Method 2. As shown in Table 1, AComm, ACA A1, PComm, L-PCA P1, L-PCA P2, and BA showed statistically significant associations, while MCA M1, R-PCA P1, R-PCA P2, and ICA were not statistically significant (*p* > 0.05). This implies that the choice of a path-finding method affected the correct detection of paths in AComm, ACA A1, PComm, L-PCA P1, L-PCA P2, and BA. The same was true in the association of Method 1 with Method 3. However, there was no statistically significant association between Method 2 and Method 3, except for AComm.

The seeded region-growing algorithm was advantageous because it did not segment unwanted vessels such as veins or outer intracranial vessels. The inclusion of the veins or outer intracranial vessels can obscure the visual appearances of the CoW arteries (Supplementary Figure S3) and make it challenging to locate the two endpoints when identifying a vessel segment in the CoW. The undetected paths in Table 1 resulted from a non-existent binary vessel mask possibly due to either under-segmentation of the seeded region-growing algorithm or non-enhancement of the vessel itself, and thus the path-finding algorithms were not able to be applied to the vessel segments. AComm had 16 undetected paths (i.e., 26.7% of the total subjects). R-PComm and L-PComm had 46 and 42 undetected paths (i.e., 76.7% and 70.0% of the total subjects), respectively.

Computational time was compared among the three methods in all subjects’ data (Supplementary Table S1). To isolate the path-finding algorithms from the manual annotation, we loaded the text file which contained all the manually annotated endpoints of the vessel segments, and we focused on measuring time on the path-finding procedures subject by subject. Method 1 took an average (±standard deviation) of 1489.4 (±191.4) ms. Method 2 took shorter than Method 1 with an average (±standard deviation) of 458.2 (±63.4) ms. Method 3 took an average (±standard deviation) of 458.0 (±63.4) ms, which was comparable to Method 2. The difference between Method 1 and Method 2 (or Method 3) was statistically significant (*p* < 0.0001), while the difference between Method 2 and Method 3 was not statistically significant (*p* > 0.4). In Methods 2 and 3, the conversion of the skeleton to the graph structure was the main bottleneck and took 98.95% and 99.00% of the total computational time, respectively. Moreover, it is important to note that manual annotation of the endpoints required the user to rotate and zoom in and out of the 3D skeletal vessel for correct identification of the vessel segments and took approximately 10 min per subject.

## 4. Discussion

**Figure 3:**
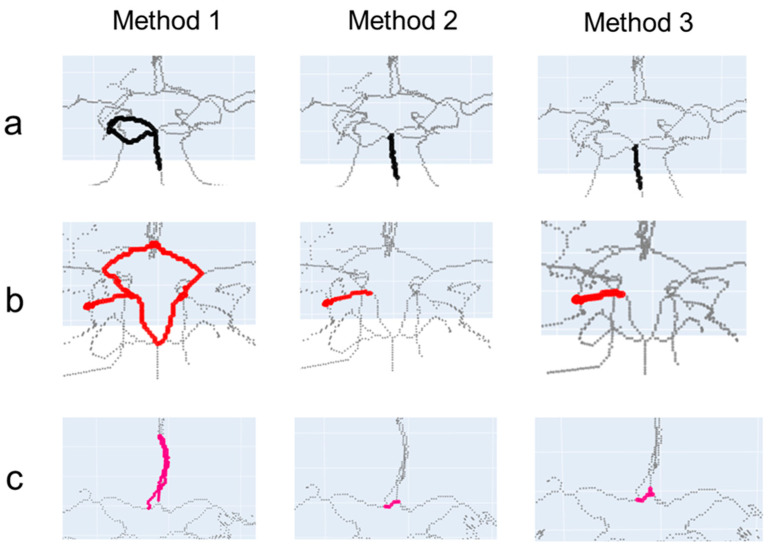
Incorrect path-finding results using Method 1 when compared with correct path-finding results using Methods 2 and 3. (**a**) Basilar artery (BA) segment, (**b**) right posterior cerebral artery P2 (R-P2) segment, and (**c**) anterior communicating artery (AComm) segment. Method 1 finds detoured paths in (**a**–**c**). The colorful lines indicate the specific vessel segments (see Figure 2 for the correspondences between colors and vessel segments).

Path finding is essential for annotating and quantifying vessel segments and has the potential to be useful in planning invasive procedures such as mechanical thrombectomy. This current study compared the performance of three path-finding algorithms in the total 840 arterial segments in the CoWs. Method 1 does not require a conversion of the skeleton’s centerlines to the graph representation and is based on the DFS algorithm. Since it does not construct a graph representation, it is deemed as rather simple, when compared to Methods 2 and 3, which require the construction of a graph and utilize the shortest path-finding Dijkstra and A* algorithms, respectively. Our path-finding results indicate that Method 1 is prone to errors when there is a loop in the vessel’s centerlines (Figure 3). The shortest path-finding algorithms can avoid such errors in case of the existence of a loop.

**Figure 4:**
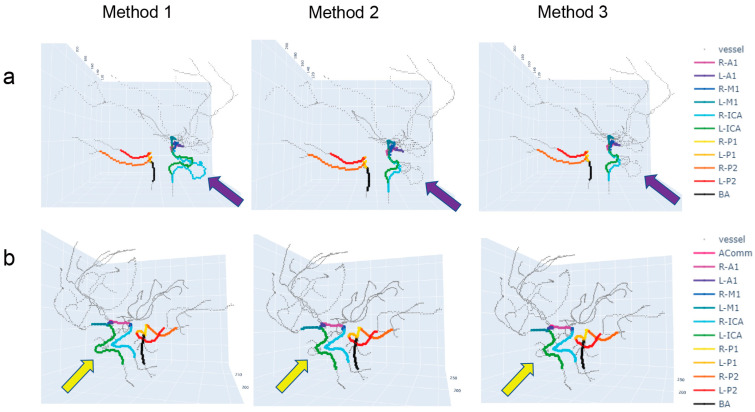
Incorrect path-finding results using Methods 2 and 3 when compared with correct path-finding results using Method 1. (**a**) The R-ICA and L-ICA segments in Method 2 found the shortest paths, but the paths are incorrect, given the nature of tortuous vessels in the ICA in general. Compare the ICA vessels indicated by the purple arrows. (**b**) The L-ICA segment in Methods 2 and 3 found the shortest paths, which is incorrect. Compare the ICA vessels indicated by the yellow arrows.

There are two possible reasons for the incorrect path-finding results. First, when the path forms a loop, Method 1 may not find the shortest path and instead can take a path that leads to the other endpoint regardless of the path’s length. The ‘loop’ path was frequent in the AComm segments, where there are alternative paths that detour via A2 segments. Second, when there are multiple paths between the two endpoints, Method 2 always finds the shortest path, which is sometimes not the correct path (Figure 4). We often detected an incorrect path by comparing it with a symmetrical vessel anatomy located in the other hemisphere of the brain. Additional routes in the main arteries may be attributed to either incorrect segmentation due to noise in the image or errors in the skeletonization process. By improving segmentation performance or denoising the gray-scale TOF-MRA images, the incorrect path-finding problem may be resolved.

Compared to the A* algorithm, Dijkstra’s algorithm finds the shortest paths for all nodes, although in our case we need only a single shortest path between the initial and destination nodes. Hence, the Dijkstra algorithm may not be the most time-efficient for path finding by nature. Since the A* algorithm only cares about finding the shortest path between the two endpoints, it is theoretically more time-efficient than the Dijkstra algorithm. However, in our evaluation, there were little or no noticeable improvements in the A* algorithm over the Dijkstra algorithm in terms of computational time, which was not expected given the inherent advantage in time efficiency in the A* algorithm.

The seeded region-growing algorithm used in this study provided under-segmentation results. This under-segmentation occurred dominantly in the PComm segments. Since we focused on the evaluation of the path-finding methods, it is a fair comparison, although there was an under-segmentation issue in several vessel segments. However, there is room for improvement in automatic segmentation. One way would be to use advanced segmentation methods based on encoder–decoder deep convolutional neural network architectures [30,31] or a multiscale image analysis approach [32]. Our study was based on MRA images, but it is possible to extract vessel segments from CTA images. Notably, a direct application of our region-growing segmentation to CTA images would be challenging because the region-growing method is based on the similarity of intensity in the blood, and contrast-enhanced arteries may not be distinguished from nearby bone structures in CTA images [33,34]. The errors in vessel segmentation are likely to occur especially in the regions where the arteries and bones are very close (e.g., internal carotid arteries, vertebral arteries, etc.). For CTA images, recent studies proposed automatic deep learning-based vessel segmentation methods to improve the accuracy of vessel segmentation [18,35].

Manual annotation of the endpoints in 3D space is tedious and can be prone to errors if not carefully checked. As such, it would be intriguing to investigate the feasibility of automatic landmark localization methods [36,37,38] to localize the vessel segments’ endpoints which we manually annotated in this study. Landmark localization of the two endpoints may also be helpful in automatically detecting occlusions of the arterial segments in ischemic stroke patients’ data.

This study has several limitations. First, the number of subjects used for the analysis is small. However, increasing the number of subjects would not significantly affect the study outcome, as there are already more than 10 segments for each subject’s MRA image data. Second, we did not perform intrarater or interrater variability in evaluating the correctness of the paths. Third, the image segmentation quality has imperfections since it showed under-segmentation results, especially in the PComm segments. However, improving the segmentation results may not significantly affect the outcome of the study because all three methods used the same segmented binary vessel masks for the analysis. Fourth, we did not consider disconnected vessel segments, which may result from the occlusions or severe stenoses of arterial segments. The development of a method for identifying disconnected vessel segments would be important for automatically detecting the vessel’s occlusion. Last but not least, we only considered arterial segments in the CoW arteries. Extending the analysis to other intracranial vessel segments such as ACA A2 and MCA M2 would be an important venue for further research.

## 5. Conclusions

Our study indicates that a graph representation of the centerlines of cerebral arteries in the CoW is advantageous in semi-automatically labeling the cerebral arteries when it is used with the shortest path-finding algorithms such as the Dijkstra algorithm and the A* algorithm. Among the three path-finding algorithms, the Dijkstra algorithm resulted in the highest accuracy of 97.1%, and it was slightly higher in accuracy than the A* algorithm. When the DFS approach was used, the incorrect path-finding results mostly occurred for the vessel segments with multiple paths connecting the two endpoints. Path-finding results for the Dijkstra and A* algorithms were incorrect when the paths were visually compared with the paths in the other brain hemisphere. Full automatization of vessel segmentation and landmark localization with deep learning would be desirable in order to increase time efficiency and avoid manual annotation procedures.

## Figures and Tables

**Table 1:** Evaluation of the path-finding methods.

		Method 1 (DFS Algorithm)		Method 2 (Dijkstra Algorithm)		Method 3 (A* Algorithm)		*p*-Value *	*p*-Value †	*p*-Value ‡	No. of Undetected Paths	Total
		No. of Correct Paths	No. of Incorrect Paths	No. of Correct Paths	No. of Incorrect Paths	No. of Correct Paths	No. of Incorrect Paths					
AComm		19	25	44	0	36	8	<0.001	<0.001	0.006	16	60
ACA A1	R	51	9	60	0	60	0	0.003	0.003	1	0	60
	L	49	10	59	0	59	0	0.001	0.001	1	1	60
MCA M1	R	55	5	60	0	60	0	0.057	0.057	1	0	60
	L	58	2	60	0	59	1	0.496	1	1	0	60
PComm	R	8	6	14	0	14	0	0.016	0.016	1	46	60
	L	10	8	18	0	18	0	0.003	0.003	1	42	60
PCA P1	R	52	8	57	3	58	2	0.204	0.095	1	0	60
	L	51	9	60	0	60	0	0.003	0.003	1	0	60
PCA P2	R	55	5	60	0	60	0	0.057	0.057	1	0	60
	L	51	9	60	0	60	0	0.003	0.003	1	0	60
BA		53	7	60	0	60	0	0.013	0.013	1	0	60
ICA	R	51	9	50	10	50	10	1	1	1	0	60
	L	51	9	52	8	52	8	1	1	1	0	60
Total		614	121	714	21	706	29				105	840

* Comparison between Method 1 (DFS algorithm) and Method 2 (Dijkstra algorithm). † Comparison between Method 1 (DFS algorithm) and Method 3 (A* algorithm). ‡ Comparison between Method 2 (Dijkstra algorithm) and Method 3 (A* algorithm).

## Data Availability

The data presented in this study are available on request from the corresponding author.

## Supplementary Materials

The following supporting information can be downloaded at: https://www.mdpi.com/article/10.3390/tomography9040113/s1, Figure S1: A slice used for the selection of three seed points. The cross-sections of the right internal carotid artery (orange), the left internal carotid artery (blue), and the basilar artery (green) are shown. The seed locations for seeded region growing are indicated by the arrows; Figure S2: The pseudo-code of the depth first search (DFS) based maze solving algorithm. This algorithm is referred to as Method 1; Figure S3: Seeded region growing. (a) A vessel segmentation result after the Otsu’s thresholding. Disconnected vessels are present, which sometimes obscure the view of the artery of interests. (b) A vessel segmentation result after applying the seeded region growing algorithm to the Otsu-thresholded vessel. Disconnected vessels disappear, and vessel visualization is improved; Table S1: Comparison of computational time measurements.
