# Supplementary material for: Effects of Path-Finding Algorithms on the Labeling of the Centerlines of Circle of Willis Arteries
Source: Tomography. 2023 Jul 24;9(4):1423–33. doi: 10.3390/tomography9040113 (PMC10366843; doi:10.3390/tomography9040113)
Supplement: Supplementary file 1 [file tomography-09-00113-s001.zip › tomography-2433546-supplementary.pdf]

Supplementary Material

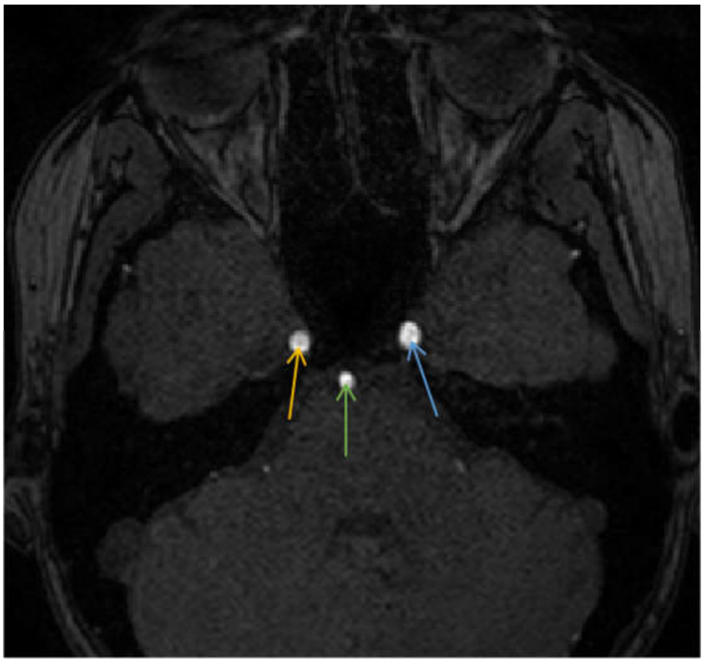

Figure S1. A slice used for the selection of three seed points. The cross-sections of the right internal carotid artery (orange), the left internal carotid artery (blue), and the basilar artery (green) are shown. The seed locations for seeded region growing are indicated by the arrows.

Algorithm

```
Initialize a stack with an element having the maze's entrance position and direction to north
(i.e., dir=0)

while (stack is not empty) {

   <row, col, slice, dir> = pop from the top of stack;
   while (dir <= 26) {
      if ((nextRow==EXIT_ROW) && (nextCol==EXIT_COL) && (nextSlice==EXIT_SLICE)) {
         terminate with success;
      if ((maze[nextRow][nextCol][nextSlice]==0) && (mark[nextRow][nextCol][nextSlice]==0){
         mark[nextRow][nextCol][nextSlice]=1;   // visited
         push <row, col, slice, dir++> to the top of stack;
         row = nextRow;
         col = nextCol;
         slice = nextSlice;
         dir = 0;
      } else
         dir++;
   }
}

printf("No path found");
```

Figure S2. The pseudo-code of the depth first search (DFS) based maze solving algorithm. This algorithm is referred to as Method 1.

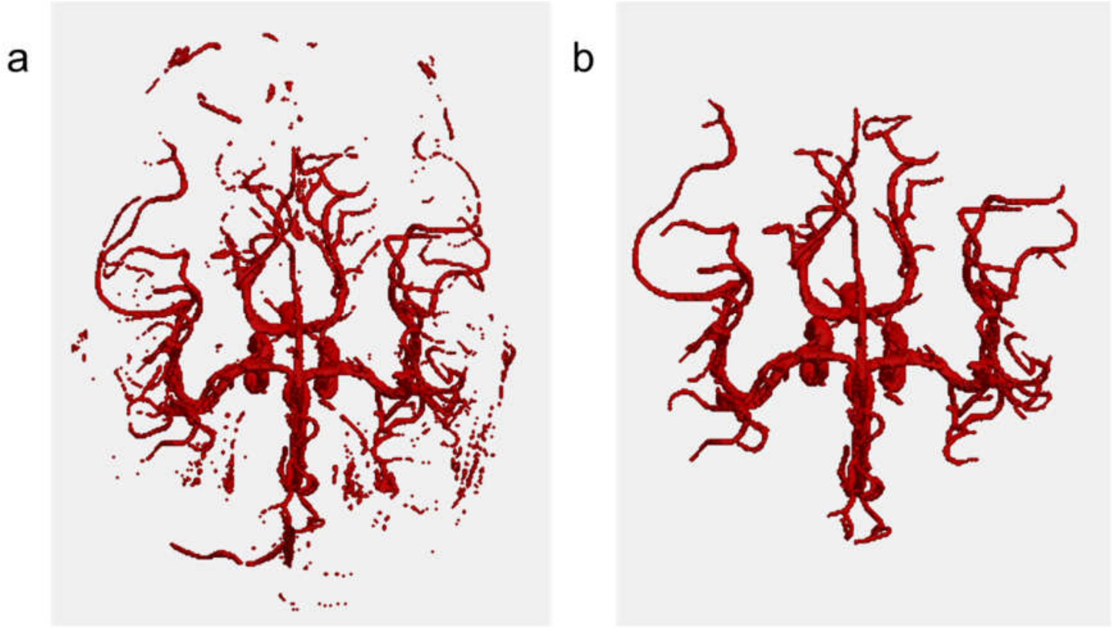

Figure S3. Seeded region growing. (a) A vessel segmentation result after the Otsu's thresholding. Disconnected vessels are present, which sometimes obscure the view of the artery of interests. (b) A vessel segmentation result after applying the seeded region growing algorithm to the Otsu-thresholded vessel. Disconnected vessels disappear, and vessel visualization is improved.

Table S1. Comparison of computational time measurements.

Subj no.	Method 1	Method 2			Method 3		
	DFS (ms)	Graph generation (ms)	Dijkstra (ms)	Graph + Dijkstra (ms)	Graph generation (ms)	A* (ms)	Graph + A* (ms)
1	1518.3	643.6	4.3	647.9	643.6	4.4	648.0
2	1536.7	424.5	6.8	431.3	424.5	4.5	429.0
3	1410.9	411.2	3.4	414.6	411.2	4.6	415.8
4	1416.8	406.3	3.6	409.9	406.3	3.4	409.7
5	1401.0	419.9	3.1	423.0	419.9	3.1	423.0
6	1404.4	410.9	3.4	414.3	410.9	3.6	414.5
7	1532.1	407.5	4.7	412.2	407.5	4.8	412.3
8	1519.8	414.4	4.4	418.8	414.4	4.3	418.7
9	1407.8	405.1	3.8	408.9	405.1	3.7	408.8
10	1413.3	408.2	3.7	411.9	408.2	3.6	411.8
11	1391.1	423.5	2.9	426.4	423.5	3.5	427.0
12	1488.3	398.1	3.8	401.9	398.1	3.6	401.7
13	1607.4	427.3	5.2	432.5	427.3	4.3	431.6
14	1370.4	410.4	4.0	414.4	410.4	3.4	413.8
15	1286.0	395.4	2.5	397.9	395.4	2.8	398.2
16	1382.5	408.3	4.0	412.3	408.3	3.1	411.4
17	1485.0	476.0	6.9	482.9	476.0	4.8	480.8
18	1425.3	460.9	3.8	464.7	460.9	4.2	465.1
19	1425.5	426.5	2.7	429.2	426.5	3.7	430.2
20	1648.5	420.0	6.6	426.6	420.0	4.6	424.6
21	1425.8	482.6	3.2	485.8	482.6	4.0	486.6
22	1284.4	440.7	3.9	444.6	440.7	3.9	444.6
23	1286.3	440.9	3.5	444.4	440.9	3.5	444.4
24	1302.1	487.3	6.9	494.2	487.3	4.7	492.0
25	1331.0	457.5	2.8	460.3	457.5	4.9	462.4
26	1372.9	470.6	4.6	475.2	470.6	5.0	475.6
27	1291.3	415.9	4.8	420.7	415.9	5.8	421.7
28	1676.4	558.1	8.5	566.6	558.1	4.6	562.7
29	1418.8	445.8	3.8	449.6	445.8	3.7	449.5
30	1301.5	417.7	7.9	425.6	417.7	4.2	421.9
31	1385.8	403.6	5.1	408.7	403.6	4.6	408.2
32	1408.2	408.7	4.2	412.9	408.7	6.2	414.9
33	1949.4	447.7	8.0	455.7	447.7	4.3	452.0
34	1307.4	466.0	3.5	469.5	466.0	6.3	472.3
35	1418.0	432.2	3.0	435.2	432.2	4.3	436.5
36	1476.8	439.5	3.4	442.9	439.5	6.2	445.7

37	1563.4	479.0	4.8	483.8	479.0	5.8	484.8
38	1426.1	455.3	3.6	458.9	455.3	6.7	462.0
39	1509.1	441.1	3.9	445.0	441.1	2.9	444.0
40	1701.9	580.7	5.6	586.3	580.7	5.7	586.4
41	1568.8	464.1	4.1	468.2	464.1	5.9	470.0
42	1459.0	438.9	3.6	442.5	438.9	5.2	444.1
43	1324.3	464.4	2.7	467.1	464.4	5.5	469.9
44	1705.1	450.9	6.4	457.3	450.9	5.4	456.3
45	1440.9	468.6	4.5	473.1	468.6	4.5	473.1
46	1550.0	435.4	4.4	439.8	435.4	5.2	440.6
47	1688.1	425.4	5.0	430.4	425.4	5.9	431.3
48	1559.3	709.3	6.9	716.2	709.3	4.5	713.8
49	1543.5	445.8	4.3	450.1	445.8	5.2	451.0
50	1395.5	433.3	3.4	436.7	433.3	5.1	438.4
51	1416.7	428.8	2.9	431.7	428.8	4.8	433.6
52	1525.1	418.4	8.4	426.8	418.4	5.8	424.2
53	1617.6	703.2	3.4	706.6	703.2	6.1	709.3
54	2007.8	427.7	10.1	437.8	427.7	3.7	431.4
55	1403.9	439.3	3.5	442.8	439.3	4.3	443.6
56	1302.8	473.8	3.6	477.4	473.8	4.8	478.6
57	2456.7	442.6	10.9	453.5	442.6	6.7	449.3
58	1583.7	473.6	11.1	484.7	473.6	6.9	480.5
59	1510.5	456.1	5.6	461.7	456.1	4.6	460.7
60	1397.7	435.0	5.6	440.6	435.0	3.9	438.9
Mean	1489.4	453.4	4.8	458.2	453.4	4.7	458.0
Std	191.4	63.1	2.0	63.4	63.1	1.0	63.4
